# Demonstration of a roll-to-roll-configurable, all-solution-based progressive assembly of flexible transducer devices consisting of functional nanowires on micropatterned electrodes

**DOI:** 10.1038/s41598-023-38635-3

**Published:** 2023-07-24

**Authors:** Inhui Han, Jungkeun Song, Kwangjun Kim, Hyein Kim, Hyunji Son, Minwook Kim, Useung Lee, Kwangjin Choi, Hojae Ji, Sung Ho Lee, Moon Kyu Kwak, Jong G. Ok

**Affiliations:** 1 Department of Mechanical and Automotive Engineering, Seoul National University of Science and Technology, 232 Gongneung-Ro, Nowon-Gu, Seoul, 01811 Republic of Korea; 2 Department of Mechanical Engineering, Dong-A University, 37 Nakdong-Daero 550-Gil, Saha-Gu, Busan, 49315 Republic of Korea; 3 School of Mechanical Engineering, Kyungpook National University, 80 Daehak-Ro, Buk-Gu, Daegu, 41566 Republic of Korea; 4 Ncoretechnology Inc., 80 Daehak-Ro, Buk-Gu, Daegu, 41566 Republic of Korea

**Keywords:** Engineering, Nanoscience and technology

## Abstract

We demonstrate continuous fabrication of flexible transducer devices consisting of interdigitated (IDT) Ag microelectrodes interconnected by ZnO nanowires (ZNWs), created via serially connected solution-processable micro- and nanofabrication processes. On an Ag layer obtainable from the mild thermal reduction of an ionic Ag ink coating, the roll-to-roll-driven photolithography process [termed photo roll lithography (PRL)] followed by wet-etching can be applied to continuously define the IDT microelectrode structure. Conformal ZNWs can then be grown selectively on the Ag electrodes to interconnect them via an Ag-mediated hydrothermal ZNW growth that does not require high-temperature seed sintering. Given that all of these constitutive processes are vacuum-free and solution-processable at a low temperature, and are compatible with continuous processing onto flexible substrates, they can be eventually configured into the roll-to-roll-processable progressive assembly. Through parametric optimizations of processes consisting of the roll-to-roll-configurable, solution-based progressive assembly of nanostructures (ROLSPAN), a flexible transducer consisting of ZNW-interconnected, PRL-ed IDT Ag electrodes can be developed. This flexible architecture faithfully performs UV sensing as well as optoelectronic transduction. The ROLSPAN concept along with its specific applicability to flexible devices may inspire many diverse functional systems requiring high-throughput low-temperature fabrication over large-area flexible substrates.

## Introduction

The fabrication of micro- and nanostructured device systems has generally relied on chip-to-chip lithography and/or vacuum-assisted deposition and etching, which has recently become a roadblock limiting the high-throughput manufacturing of diverse flexible devices in areas such as sensor, electronic, energy, and healthcare industries^1–8^. For instance, UV photodetector systems employing metal-oxide (MOx) nanostructures integrated with micro- and nanopatterned electrodes have been widely used in safety, hygiene, and communication applications, among others^7–14^, yet there remains a need for more scalable and practical fabrication protocols to meet the increasing demand for low-cost flexible devices^15–18^. Exploiting alternative routes to creating micropatterned electrodes without a batch process consisting of wafer-bound photolithography, vacuum deposition, and plasma etching, and to growing MOx nanostructures without high-temperature chemical vapor deposition process, are therefore in strong demand.

To this end, we demonstrate a functional micro/nanoarchitecture consisting of solution-processed ZnO nanowire (NW) structures selectively grown on solution-processed Ag electrodes fabricated by continuous rollable photolithography for flexible transducer devices such as UV sensors. More specifically, initially we create Ag layers from the mild thermal reduction of an ionic Ag ink coating, which can be performed on any common—either rigid or flexible—substrate, including glass and polymer film^19–22^. The Ag layer can then be patterned into interdigitated (IDT) microelectrodes through continuous photo roll lithography (PRL)^23–25^; during the PRL process, a flexible photomask-attached hollow quartz roll inside of which a UV exposure source is mounted rolls over the photoresist (PR)-coated substrate fed continuously underneath. Finally, ZnO NWs (ZNWs) can be conformally grown on the PRL-processed IDT Ag electrodes to interconnect them via a low-temperature hydrothermal reaction, with the Ag surface providing favorable sites for ZnO crystal nucleation and growth^22,26,27^, thereby obviating the high-temperature sintering of a textured ZnO seed layer. As briefly introduced above and as detailed later in this paper, all of these constitutive processes involve vacuum-free and solution-based steps and are compatible with continuous processing onto flexible substrates. They can therefore be eventually configured onto a roll-to-roll-processable progressive assembly line, as is conceptually demonstrated in this work.

## Experimental methods

### Materials

An ionic Ag ink (TEC-CO-011, InkTec) was diluted (sonication for 10 min) in isopropyl alcohol (IPA), typically at a volume ratio of 1:1, followed by PTFE membrane filtering (0.2 µm pore size, ADVANTEC). This is simply referred to as an ‘ionic Ag solution’ hereafter. During the photolithographic patterning process, the following chemicals were used: hexamethyldisilazane (HMDS; AZ AD Promoter-K, Merck Electronics), negative PR (DNR-L300-40, Dongjin Semichem, Co., Ltd.), developer (AZ 300 MIF Developer, Merck Electronics), and an etchant (Al etchant type A, Transene). For the hydrothermal ZNW growth process, zinc nitrate hexahydrate (N_2_O_6_Zn·6H_2_O, reagent grade, 98%, Sigma-Aldrich) and hexamethylenetetramine (HMTA; C_6_H_12_N_4_, ACS reagent, ≥ 99.9%, Sigma-Aldrich) were mixed in deionized (DI) water.

### Development of the PRL system

The main modules of the custom-built PRL system used here consisted of a rollable UV exposure unit, a motorized moving stage assembly, and controllers (Fig. S1 in the *Supporting Information*). For the UV exposure unit, a transparent quartz tube with a diameter of 150 mm and a thickness of 3 mm was assembled with two ball bearings (Model 6832, NSK Corp, Japan) at both ends and was installed in an aluminum-profile frame. A high-power linear UV source (300–1300 mW, Linear LED series, UV SMT Corp, Korea) was placed inside the quartz tube, with its illumination slit (~ 1 mm width) directed downward. In the stage assembly, a height-adjustable microstage (SS6H-120-S, ST1 Corp, Korea) was mounted on the motorized moving stage (SM1-0830-4S, ST1 Corp, Korea) to control the gap between the substrate and the outer surface of a quartz roll. The moving stage was operated in the horizontal direction by a precise step motor (A3K-S545W, Autonics, USA) having a high resolution of 2 μm and a maximum moving speed of 20 mm/s. A ~ 2 mm-thick polydimethylsiloxane (PDMS) sheet was prepared; a 10:1 (weight ratio) mixture of an elastomer base and a curing agent (Sylgard 184, Dow Corning Corp, USA) was poured onto a glass substrate, degassed for five to ten minutes in a rotary pumped chamber, put on a horizontal table, and cured at 70 °C for one hour. The prepared thin PDMS sheet was conformally attached to the outer surface of a quartz roll to attach the photomask.

### Fabrication of the flexible photomask

A 50-nm-thick Al layer was deposited onto a polyethylene terephthalate (PET; RX000, X type, Hyosung) film pre-cleaned with acetone and IPA. A HMDS layer was then spin-coated (4000 rpm, 20 s) onto the Al-deposited PET film, followed by baking at 95 °C for one minute. A PR layer was subsequently spin-coated (4000 rpm, 20 s) onto the HMDS/Al/PET sample, followed by baking at 95 °C for 90 s. UV photolithography was then applied to the PR/HMDS/Al/PET sample using a Karl Suss MA6 instrument and a 7″ Cr/glass mask having IDT patterns of specific linewidth/interspace sizes (e.g., 5, 10, 20, and 40 µm, as will be specified in the text). This was followed by post-exposure baking (PEB) at 110 °C for 90 s. The flexible Al/PET photomask was completed through the sequential wet-bench process of PR develop (1 min), wet-etching (3–4 min) of the exposed Al part, PR strip in acetone, and IPA rinsing, and was then attached to the PDMS-wrapped surface of a quartz roll in the PRL system.

### Fabrication of an IDT Ag microelectrode by PRL

To create an Ag layer on the target substrate (typically pre-cleaned with acetone and IPA), an ionic Ag solution was spin-coated (2000 rpm, 20 s), soft-baked (90 °C, 1 min), and then hard-baked (120 °C, 5 min) as needed. The ionic Ag solution can be coated by other methods including dip-coating, simple drop casting, and ink-jetting, as previously demonstrated in the literature^19,21^. HMDS and PR layers were then sequentially coated onto the Ag-coated substrate using conditions identical to those described above. The PR/HMDS/Ag-coated substrate was brought into contact with the IDT-patterned photomask-attached quartz roll surface such that the substrate-mask contacting line was aligned to the exposure slit line of a linear UV lamp mounted inside the quartz roll. While controlling the UV power intensity (20–50% of the maximum intensity; simply referred to as ‘20% UV’ to ‘50% UV’ hereafter) and the rolling speed (1000–4000 μm/s), UV exposure onto the substrate was continuously conducted by PRL, followed by PEB at 110 °C for 90 s. After the PR develop process (30–40 s) and hard-baking (110 °C, 1 min), the substrate was immersed in the etchant and gently agitated for the wet-etching of the exposed Ag part. The IDT Ag microelectrode structure was completed by PR strip and rinsing with DI water.

### Growth of ZNW structures

The full procedure of Ag-seeded hydrothermal ZNW growth can be found in the literature^22,26,27^. Briefly, the precursor solution was initially prepared by mixing 3.75 g of N_2_O_6_Zn·6H_2_O and 1.75 g of HMTA in a beaker containing 500 ml of DI water (i.e., resulting in a concentration of ~ 25 mM for both materials) on a 140 °C hot plate for one hour under 200 rpm magnetic bar stirring. The Ag-patterned substrate was immersed in the precursor solution beaker, which was put into a bath circulator set to 90 °C for 15 h for ZNW growth. The ZNW/Ag-coated substrate was gently rinsed with DI water and was then dried by gentle N_2_ blowing.

### Characterization

A high-resolution metallurgical microscope (MIC-S39B, BEIMEINCE Corp.) was used for optical microscope (OM) imaging along with a digital analysis. A JSM-6700F field-emission (JEOL Ltd.) SEM instrument was used for scanning electron microscope (SEM) imaging at the typical operation voltage of 10 kV. A digital multimeter (Keithley DMM 6500) was used to measure the electrical resistance of the samples. For the UV-induced photocurrent measurement, a spot-type UV source (INNO-CURE 5000, Lichtzen Co., Ltd.) was used as a UV source; its power intensity was controlled in the range of 100–500 mW/cm^2^ while the distance between the UV source and the sample was fixed at 2 cm. The photocurrent generated in the sample was measured using a multimeter under DC bias applied through a Zhaoxin RXN-305D power supply in a lightproof black box having a pinhole through which the UV spotlight was illuminated.

## Results and discussion

### Concept of ROLSPAN: principle and criteria

**Figure 1:**
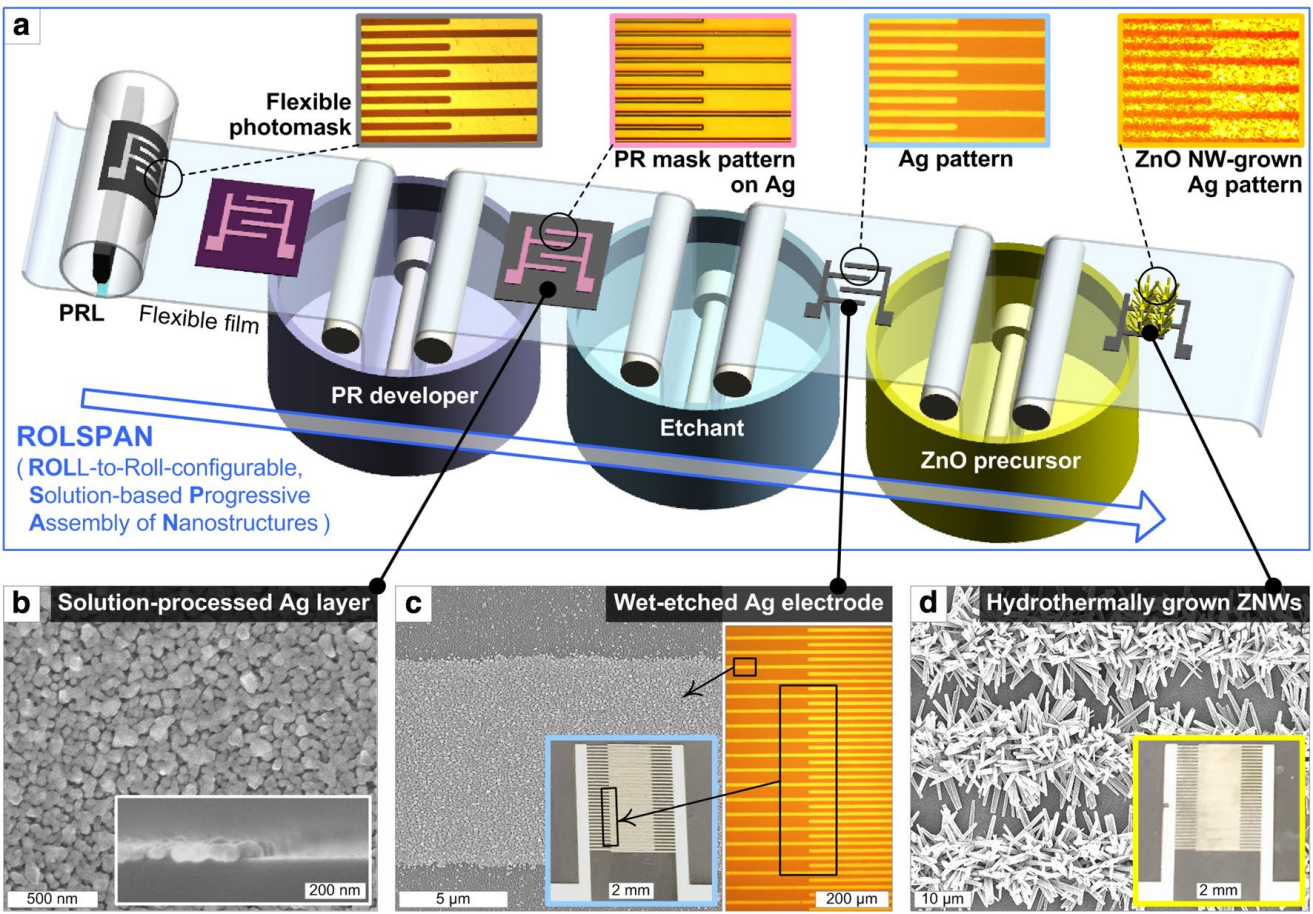
(**a**) Conceptual scheme of the overall ROLSPAN procedure comprising PRL processing for PR pattern definition on the ionic Ag solution-processed Ag layer, wet-etching fabrication of the IDT Ag microelectrode structure, and the ZNW growth thereto. The upper-right insets show OM images of each step. (**b**) Top-view SEM image of the solution-processed Ag layer, where the PR coating is applied before feeding into the PRL system. The inset shows a cross-sectional SEM image of the Ag layer. (**c**) SEM and low-magnification OM images of the IDT Ag microelectrode structure fabricated by wet-etching. (**d**) SEM image of the ZNW-interconnected IDT device. The insets in (**c**) and (**d**) show corresponding optical photographs of the devices overall.

**Figure 2:**
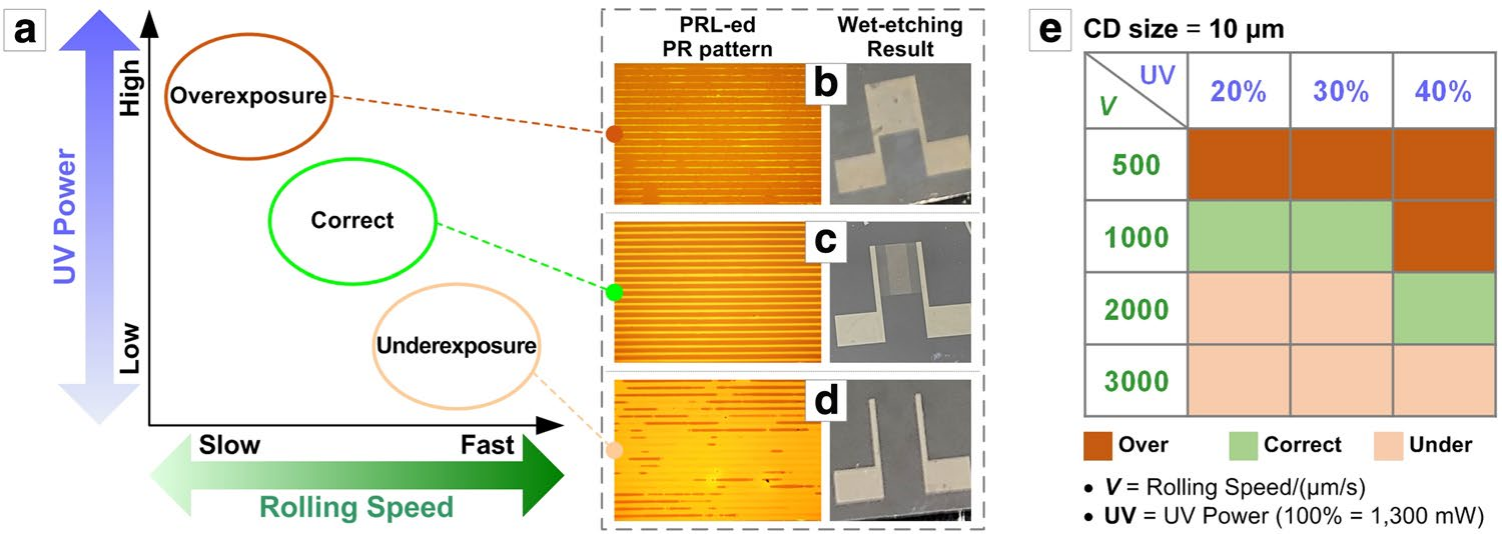
(**a**) Schematic plot of the UV exposure quality depending on the rolling speed and UV power in the PRL process. (**b**–**d**) OM images of typical PR mask pattern shapes (left-side images; dark part is PR) and optical photographs of the resulting IDT SPAN microelectrode structures (right-side images) for the (**b**) overexposure, (**c**) correct, and (**d**) underexposure cases. (**e**) Tabulated PRL parameters for each exposure condition for the 10 µm IDT pattern.

**Figure 3:**
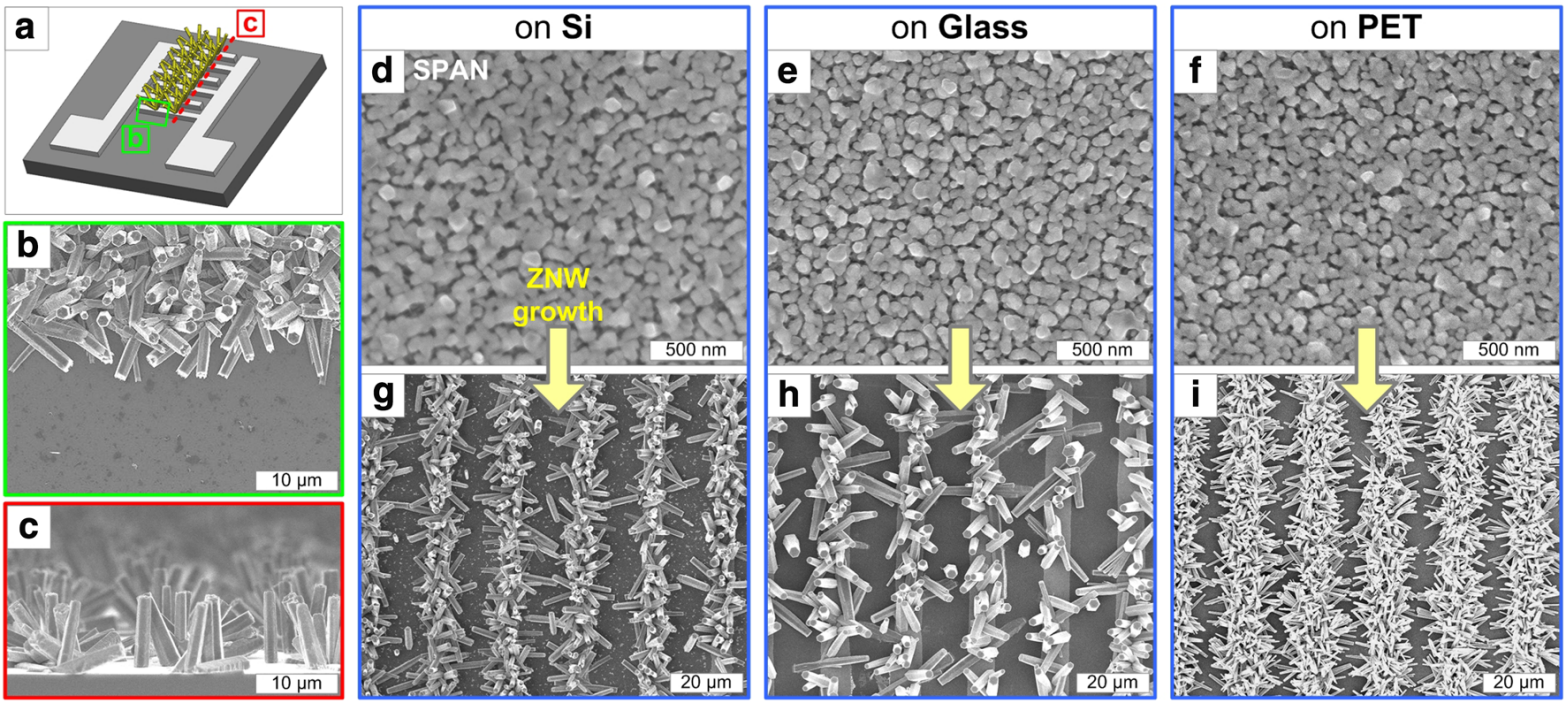
(**a**) Scheme of the ZNW-grown IDT SPAN device. (**b**) SEM image of ZNWs selectively grown on the SPAN area near the boundary of the bare substrate area. (**c**) Cross-sectional SEM image of ZNW-grown IDT pattern lines. SEM images of SPAN layers prepared on (**d**) Si, (**e**) glass, and (**f**) PET substrates, and (**g**–**i**) ZNWs grown on IDT SPAN structures fabricated on these substrates.

Figure 1 presents the conceptual overall scheme along with OM and SEM images of the representative step-wise results of the roll-to-roll-configurable, solution-based progressive assembly of nanostructures (ROLSPAN), which consists of three main processing steps: (1) PRL for microelectrode-shaped PR patterning (Fig. 1a) on an ionic Ag solution-processed Ag layer (Fig. 1b), (2) wet-etching for the fabrication of an IDT Ag microelectrode structure (Fig. 1c), and (3) Ag-seeded hydrothermal ZNW growth for the fabrication of a ZNW-based sensor device (Fig. 1d). All of these steps are solution-processable, free from the need for high-temperature and/or vacuum treatments, and compatible with continuous processing onto a large-area flexible substrate. As part of the concept of ROLSPAN, a flexible film can be conveyed in a progressive assembly line using support rolls over solution baths and bearing-supported, dumbbell-like rolls (to protect the fabricated structure from direct contact with the roll) in the solutions. While the methods to control the thickness, morphology, and properties of the ionic solution-processable Ag nanostructure (SPAN) have been previously studied in detail^20^, the SPAN film used in this study (Fig. 1b) has typical thickness and sheet resistance values of ~ 50–55 nm and ~ 4–5 Ω/□, respectively, suitable for the ROLSPAN process. In PRL, uniform UV exposure through a curved quartz roll onto the PR-coated SPAN substrate under conformal contact and the definition of an adequate UV dose by controlling the rolling speed and UV intensity would be two important aspects. The linewidth of the IDT patterns would also affect the PRL- and wet-etching-driven microelectrode fabrication quality. During ZNW growth, the interspaces of the IDT patterns and the ZNW growth time would determine how many ZNWs having proper aspect ratios interconnect the two IDT electrode members to tune the sensing characteristics. Successful ROLSPAN, toward many functional flexible devices, would be achieved by finding and sequentially combining the proper conditions for these features.

### Parametric control and optimization of the PRL fabrication of IDT SPAN electrodes

Because PRL proceeds by the continuous frictional conveyance of a substrate driven by the rolling of a UV-mounted quartz roll, achieving conformal contact between the roll and the substrate is one of the most important aspects. A thin PDMS sheet having a low Young’s modulus (~ 1.8 MPa^28^) plays a versatile role here; usually cut larger than the photomask size, it not only helps firmly and conformally to attach the flexible photomask onto the quartz roll surface but also facilitates conformal contact between the photomask and the substrate. It additionally provides a cushion under pressure to prevent the hollow quartz tube from breaking. Another significant consideration is to secure uniform UV exposure onto the planar substrate surface while using the UV source mounted inside a hollow quartz cylinder. Here, the UV light may diverge along the quartz roll’s circumferential direction. This issue can be resolved by the installation of a narrow slit in front of the UV source. The effect of a slit can be mathematically determined by the following equation (Eq. 1):

1 $$\uptheta =2\mathrm{w}/\mathrm{D},$$

where θ is the central angle of the arc, w is the arc length, and D is the diameter of a cylinder, as also schematically expressed in Fig. S2a in the Supporting Information. In this experiment, θ, w, and D correspond to the UV light diverging angle, UV exposure width, and the diameter of the quartz roll, respectively. When using a slit of w = 1 mm and a quartz roll of D = 150 mm, according to Eq. (1), the light-diverging angle can be calculated to be 0.0133 rad, which confirms that the curvature of a quartz roll has a negligible effect on uniform UV exposure through the slit to the substrate surface. As will be characterized below, the critical dimension (CD; for the linewidth and interspace at a 1:1 width ratio) of the IDT micropattern is systematically varied at 5, 10, 20, and 40 µm in this study; a drawing of the full design and dimensions of the IDT micropattern is presented in Fig. S2b in the Supporting Information. As a reference, the application of a wider slit up to w = 2.5 mm allows PRL for larger micropatterns (i.e., CD of 20 µm or larger) with acceptable quality. Nonetheless, we kept w = 1 mm, especially for the uniform PRL fabrication of finer micropatterns (i.e., CD of 10 µm or smaller) as this value was found to be more suitable eventually for the subsequent ZNW-interconnecting process, which will be discussed later in this paper.

Once the PRL system ensures conformal contact between the photomask and the substrate and uniform UV exposure through the slit, we can investigate the optimal UV exposure dose, which is controllable by two parameters in PRL: the rolling speed and the UV intensity^23,29,30^. The UV dose to the substrate surface per unit time should generally increase at a lower rolling speed and higher UV intensity. More specifically, the PRL result could be divided largely into three regions in the rolling speed–UV power coordinate: over-, proper, and underexposure, as schematically plotted in Fig. 2a. Overexposure or underexposure, stemming from ill-controlled rolling speed and/or UV intensity during PRL, can result in ill-defined IDT SPAN electrodes after the PR develop and wet-etching steps; for the negative PR layer used in this study, an overexposed PR mask could cause insufficient etching of the IDT interspaces (Fig. 2b), while an underexposed PR may not effectively mask the IDT lines in the etchant (Fig. 2d). The proper UV exposure time, obtainable based on an adequate rolling speed and proper UV intensity level, can lead to a well-defined IDT SPAN microelectrode structure (Fig. 2c). The optimal UV dose can be tuned depending on the pattern dimension. Faithful IDT SPAN patterns with CD sizes of 5, 10, 20, and 40 µm could be fabricated at specific rolling speeds and UV intensity levels; Fig. 2e exemplifies these ‘correct’ PRL conditions for an IDT pattern with a CD size of 10 µm (i.e., both the linewidth and interspace of 10 µm, simply referred to as the ‘10 µm IDT structure’ hereafter). Full datasets for other CD sizes are tabulated in Fig. S3a–c along with the corresponding exemplary results (Fig. S3d) in the *Supporting Information*. It is clear, however, that all IDT structures follow the general trend shown in Fig. 2a. Here, we utilized a rolling speed of 1000 µm/s and 20% UV intensity as the optimal UV exposure condition for IDT SPAN structure fabrication, unless otherwise specified.

Although a single photomask was used in this study, the PRL process can also be applied to overlay lithography using multiple photomasks in order for the fabrication of more complex, multi-level structures. In this case, the alignment between photomasks is an important issue in common with the normal photolithography. Here the laser- or LED-assisted alignment technique developed for roll-to-roll imprinting^31^ or printing^32,33^ can be utilized in the overlay PRL process; its positional accuracy can be improved by the optical monitoring of alignment key marks through the transparent photomask.

### Ag-mediated ZNW growth on IDT SPAN electrodes

Conformally oriented, high-density ZNWs can be grown selectively on a SPAN surface via the Ag-mediated ZnO crystal growth mechanism suggested in the literature^22^, with few grown on the surrounding substrate region (see Fig. 3b). Applied to the IDT SPAN structure, this Ag-mediated ZNW growth process can tactfully induce ZNWs to ‘bridge’ the two IDT electrode members, as schematically illustrated in Fig. 3a. Figure 3d–i demonstrate the results of ZNW growth on IDT SPAN structures fabricated on various substrates. The typical diameter and length of the ZNWs grown in this experiment were 750–800 nm and 7–8 µm, respectively, as indicated in Fig. 3c; the diameter and aspect ratio of ZNWs can be tailored by controlling the SPAN morphology and hydrothermal growth temperature and time^22,26,27^. Given that the Ag-mediated hydrothermal ZNW growth process obviates the high-temperature seed preparation process (e.g., ZnO seed sintering at 350 °C), ZNWs can be grown at a low temperature on diverse substrates, including a Si wafer, glass, and flexible polymers such as PET, as exemplified in Fig. 3g–i. The ZNW morphology appears slightly different depending on the substrate materials; this may be related to the substrate properties including the surface energy and micro- and nanoscale topography as well as the initial SPAN morphology, which is indeed under detailed investigation for a specialized report.

Recalling that the ROLSPAN concept particularly targets flexible devices, we focus on the PET substrate; additionally, PET is apparently more favorable for growing ZNWs with higher density levels compared to Si and glass in this experiment. Considering the aforementioned typical ZNW geometry, here we could determine that the 10 µm IDT structure is the best candidate for ‘ZNW bridging’. On the other hand, few 7–8 µm-long ZNWs could interconnect the electrodes of the 20 µm and 40 µm IDT structures. For the 5 µm IDT structure, the SPAN line pattern area may be too narrow to accommodate a sufficient number of ZNWs for reliable functioning; additionally, the difficulty of PRL fabrication in this case could increase due to the smaller CD size. As another concern for flexible device development, the good adhesion of SPAN electrodes should be secured. Indeed, we have demonstrated that the SPAN layer exhibits great adhesion to general substrates including flexible plastics such as PET and PI, in our previous study^20^. This allows the change in the SPAN’s electrical resistance upon mechanical bending of the flexible substrate to stay within the controlled and reversible range^20,22^. The ZNW-bridged IDT SPAN structure is thus promising for flexible transducers as will be discussed in the later part of this paper.

### Characterization of a ZNW-bridged IDT SPAN device as a flexible UV sensor

The ZNW-interconnected 10 µm IDT SPAN structure fabricated on a PET substrate (simply referred to as the ‘ZNW/IDT device’ hereafter) and/or its fabrication concept can be utilized in many practical device systems. Here, we demonstrate one specific functional application of the ZNW/IDT architecture: a flexible UV sensor. Upon illumination of UV light, excitons are generated and separated into electrons and holes inside the n-type semiconductor ZnO. The holes are shortly trapped at the ZnO surface but the electrons as free charge carriers are collected at the electronic circuitry^34–36^. Likewise, UV illumination onto a ZNW/IDT device may produce an electric current with the level to be controlled by the device resistance, applied bias, and UV intensity.

**Figure 4:**
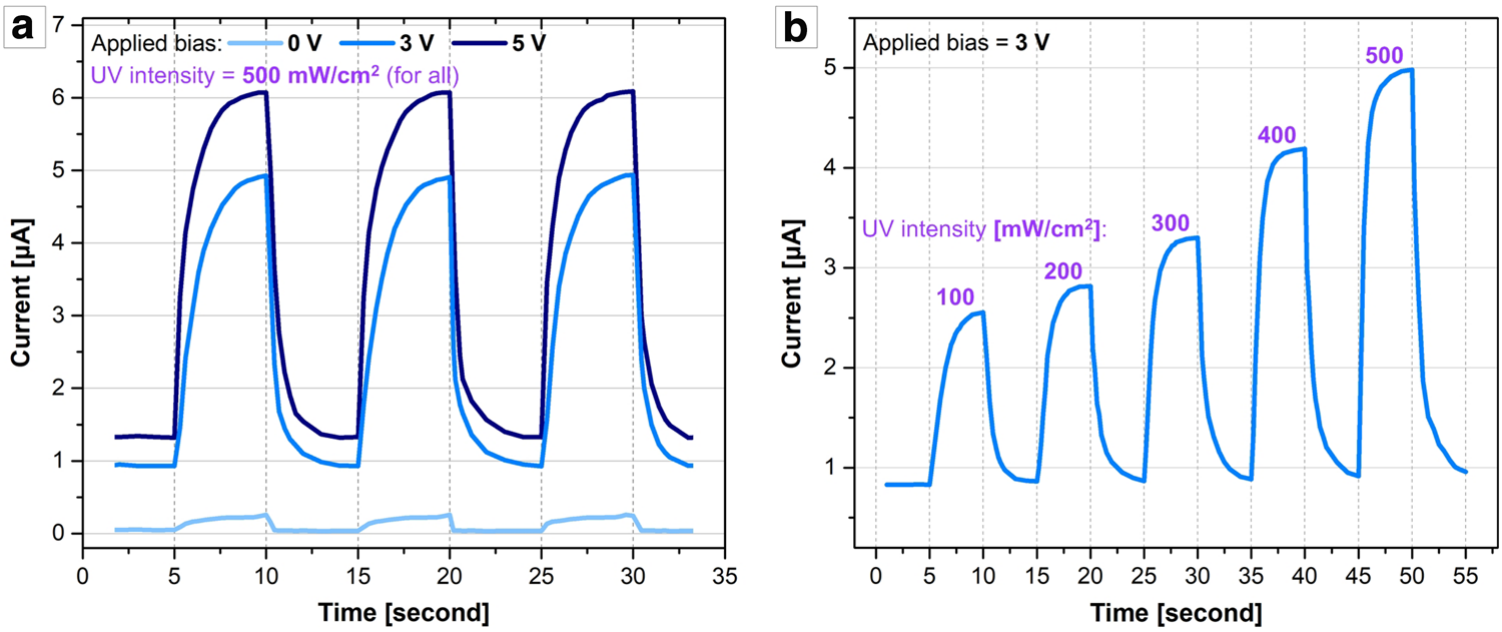
(**a**) Photocurrents (versus time) measured at various applied biases of 0 V, 3 V, and 5 V under pulsed UV illumination at a fixed intensity of 500 mW/cm^2^. (**b**) Photocurrent (versus time) measured under UV illumination of various intensity levels at 3 V bias.

We initially characterized the electric currents produced by the ZNW/IDT device upon controlled UV illumination and under controlled applied bias. Figure 4a shows electric current plots measured under 500 mW/cm^2^ UV illumination turned on and off for controlled timespans, at 0 V, 3 V, and 5 V bias. As the applied bias increases, the current value increases; the net increases in the current when the UV light is on (i.e., photocurrent) at 3 V and 5 V are ~ 4 µA and ~ 4.7 µA, respectively. It is clear that the photocurrent generates and decays with a competent response to the pulsed UV light; both the rise and decay times are less than a second under reasonable biases, which is comparable to the other ZnO/Ag-based transducer systems^37,38^. Interestingly, a measurable electric current could be transduced from the UV light energy without external bias. This indicates that the ZnO/Ag system is energetically favorable; because ZnO has a higher work function (~ 5.3 eV) than Ag (~ 4.3–4.7 eV), the ‘built-in’ potential is formed in the direction from Ag to ZnO by Fermi level alignment at the ZnO/Ag interface, as similarly studied for the ZnO/carbon nanotube (CNT) system^34,35^. The free electrons generated inside ZnO could therefore be conducted to Ag along this potential ‘slope’ without external energy. This energetically favorable ZnO/Ag architecture can thus serve as a power-efficient optoelectronic transducer for the outdoor, portable, mobile, and/or power-saving applications. Figure 4b shows the current measurement result obtained from various UV intensity levels under 3 V bias. The photogenerated current values are highly sensitive to the UV intensity, confirming that the ZnO/IDT device can function as a reliable UV sensor.

### Future perspectives for ROLSPAN: device and process

When the ZNW/IDT structure on a flexible substrate is bent, the number of ZNWs maintaining an interconnection between the two IDT electrode members can change. When bent concavely, more of the ZNWs from each side of the two IDT electrode members could make additional contact, thereby reducing the device resistance, and vice versa for convex bending. Figure S4 in the Supporting Information shows the preliminary result of a proof-of-concept experiment: resistance versus bending. Modulation of the ZNW/IDT device resistance can tune the UV-induced photocurrent value under the identical condition of UV illumination and applied bias. This encourages us to develop the bending-sensitive flexible UV transducer consisting of multiple electrodes interconnected by ZNWs. However, we may need to address several upcoming issues. First, the durability of electrodes against delamination and/or crack upon repeated mechanical deformation should be secured; one promising way is to embed the metallic wires in the engraved micropattern, as similarly presented for durable flexible heaters^39^. Second, the electrode design may be possibly modified; instead of the IDT structure, an array of separate micrograting lines can be a better candidate to enhance the bending-dependent sensitivity. Indeed, we are currently working on this interesting study, by collectively utilizing the linearly inscribed microtrench patterns^40,41^, solution-processed Ag wires embedded therein, and ZNWs selectively grown on and interconnecting those durably embedded Ag micrograting electrodes. Of course, the ROLSPAN protocol can apply to most parts of these works, too.

Finally, the continuous processability of ROLSPAN can be further improved by tuning the process parameters of PRL and nanostructure growth processes. The PR coating thickness and UV power intensity and the concentration and temperature of the developer and etchant can be modulated for smoother roll-to-roll processing during PRL and subsequent wet-etching steps. More importantly, the nanostructure growth can be much accelerated by modifying the process and device structure. For instance, once the ZnO crystals are nucleated in a ZnO precursor bath for a relatively short time, the sample can be roll-to-roll conveyed into another bath containing ionic Ag species. Here, additional UV illumination can be applied for ZnO-assisted Ag nanostructure growth, where the UV light-induced free electrons can reduce the Ag ions into the metallic Ag nanostructure on the ZnO surface without the aid of thermal energy. Indeed, we are currently developing this room-temperature photoreduction process for fabrication of Ag/ZnO nanoarchitectures, which is much faster than the typical hydrothermal growth. While a preliminary result is shown in Fig. S5 in the Supporting Information, the detailed procedure, parametric study, and characterization will be presented in a separate report. Such processes can better be adopted in the ROLSPAN fabrication.

## Conclusions

In summary, we have suggested the ROLSPAN concept for the continuous fabrication of scalable and flexible transducer devices in a roll-to-roll-configurable manner. This has been demonstrated by serially connecting the SPAN layer fabrication process, the PRL patterning of IDT SPAN electrodes, and the hydrothermal ZNW growth thereto, all of which are solution-processable at a low temperature and are thus readily applicable to flexible polymer substrates. Through parametric optimizations of those constitutive processes, we have developed the ZNW/IDT architecture, where the faithfully defined IDT SPAN electrodes are interconnected by ZNWs. The flexible ZNW/IDT structure has exhibited promising performance as a reliable energy-efficient UV sensor. Beyond the flexible ZNW/IDT transducer demonstrated in this study, the ROLSPAN fabrication concept may be feasible for developing many functional applications in a continuous and high-throughput fashion possibly by modifying, adding, or replacing the process components, including but not limited to resistive gas sensors, electromechanical transducers, and electrochemical cells.

## Supplementary Information

Supplementary Figures.

## Data Availability

The datasets used and/or analyzed in the current study are available from the corresponding author upon a reasonable request.

## Abbreviations

ZNWs: ZnO nanowires
IDT: Interdigitated
PRL: Photo roll lithography
ROLSPAN: Roll-to-roll-configurable, solution-based progressive assembly of nanostructures
MOx: Metal-oxide
NW: Nanowire
PR: Photoresist
IPA: Isopropyl alcohol
HMDS: Hexamethyldisilazane
HMTA: Hexamethylenetetramine
DI: Deionized
PDMS: Polydimethylsiloxane
PET: Polyethylene terephthalate
PEB: Post-exposure baking
OM: Optical microscope
SEM: Scanning electron microscope
SPAN: Solution-processable Ag nanostructure
CD: Critical dimension
CNT: Carbon nanotube
