# Supplementary material for: Demonstration of a roll-to-roll-configurable, all-solution-based progressive assembly of flexible transducer devices consisting of functional nanowires on micropatterned electrodes
Source: Sci Rep. 2023 Jul 24;13:11980. doi: 10.1038/s41598-023-38635-3 (PMC10366188; doi:10.1038/s41598-023-38635-3)
Supplement: Supplementary file 1 — Supplementary Figures. [file 41598_2023_38635_MOESM1_ESM.docx]

*Supporting Information* for

**Demonstration of a roll-to-roll-configurable, all-solution-based progressive assembly of flexible transducer devices consisting of functional nanowires on micropatterned electrodes**

Inhui Han^1,†^, Jungkeun Song^1,†^, Kwangjun Kim^1,†^, Hyein Kim^1^, Hyunji Son^1^, Minwook Kim^1^, Useung Lee^1^, Kwangjin Choi^1^, Hojae Ji^1^, Sung Ho Lee^2,^*, Moon Kyu Kwak^3,4,^* & Jong G. Ok^1,^*

^1^ Department of Mechanical and Automotive Engineering, Seoul National University of Science and Technology, 232 Gongneung-ro, Nowon-gu, Seoul 01811, Republic of Korea

^2^ Department of Mechanical Engineering, Dong-A University, 37 Nakdong-Daero 550-gil, Saha-gu, Busan 49315, Republic of Korea

^3^ School of Mechanical Engineering, Kyungpook National University, 80 Daehak-ro, Buk-gu, Daegu 41566, Republic of Korea

^4^ Ncoretechnology Inc., 80 Daehak-ro, Buk-gu, Daegu 41566, Republic of Korea

^†^ These authors contributed equally to this work.

*Corresponding authors:

Dr. Sung Ho Lee, sunghol@dau.ac.kr, Tel. +82-51-200-7644

Dr. Moon Kyu Kwak, mkkwak@knu.ac.kr, Tel. +82-53-950-5573

Dr. Jong G. Ok, jgok@seoultech.ac.kr, Tel. +82-2-970-9012


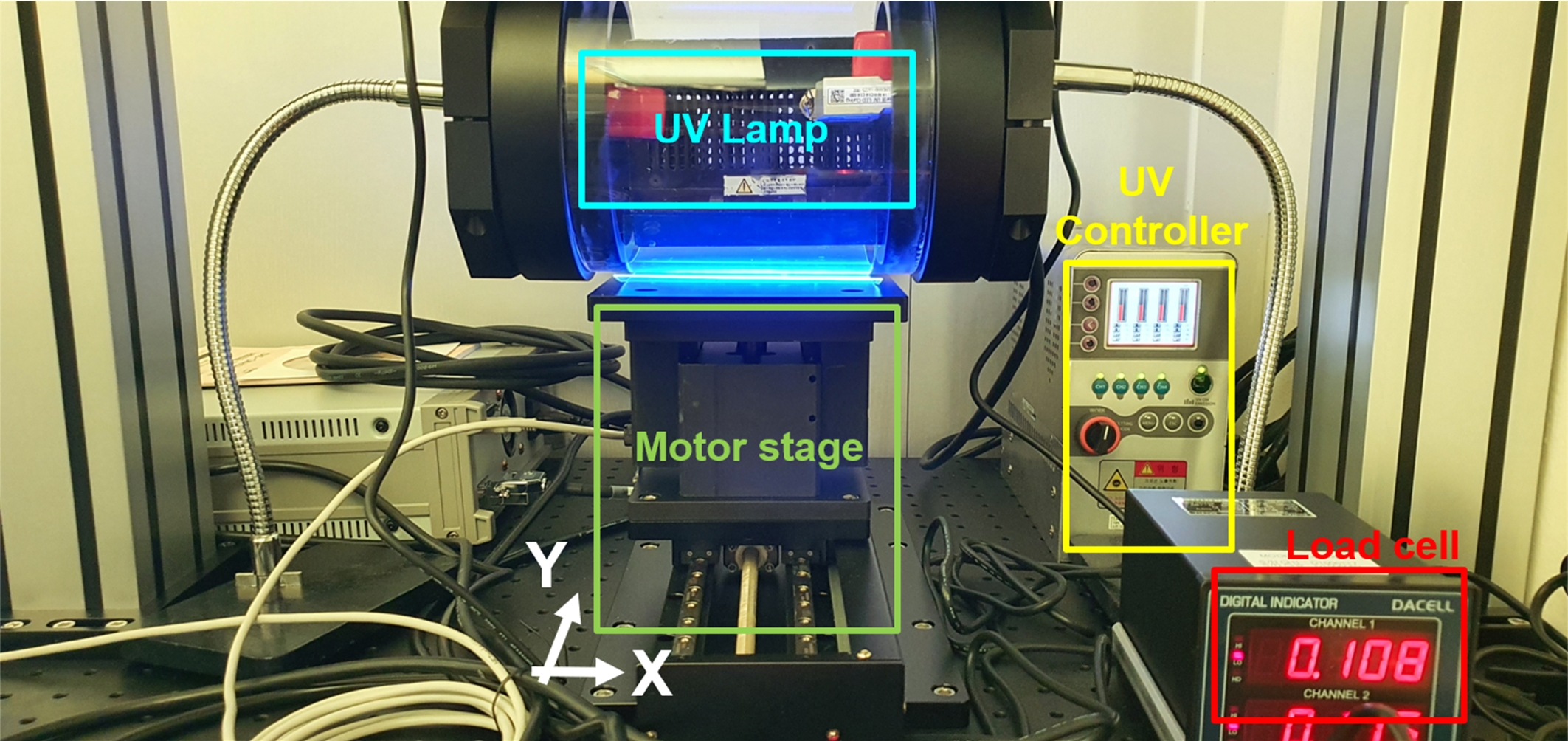


**Figure S1**. Optical photograph of the photo roll lithography (PRL) system consisting of a UV exposure unit with a slit-type UV lamp mounted inside a quartz roll, a motorized stage unit, and UV and force controllers


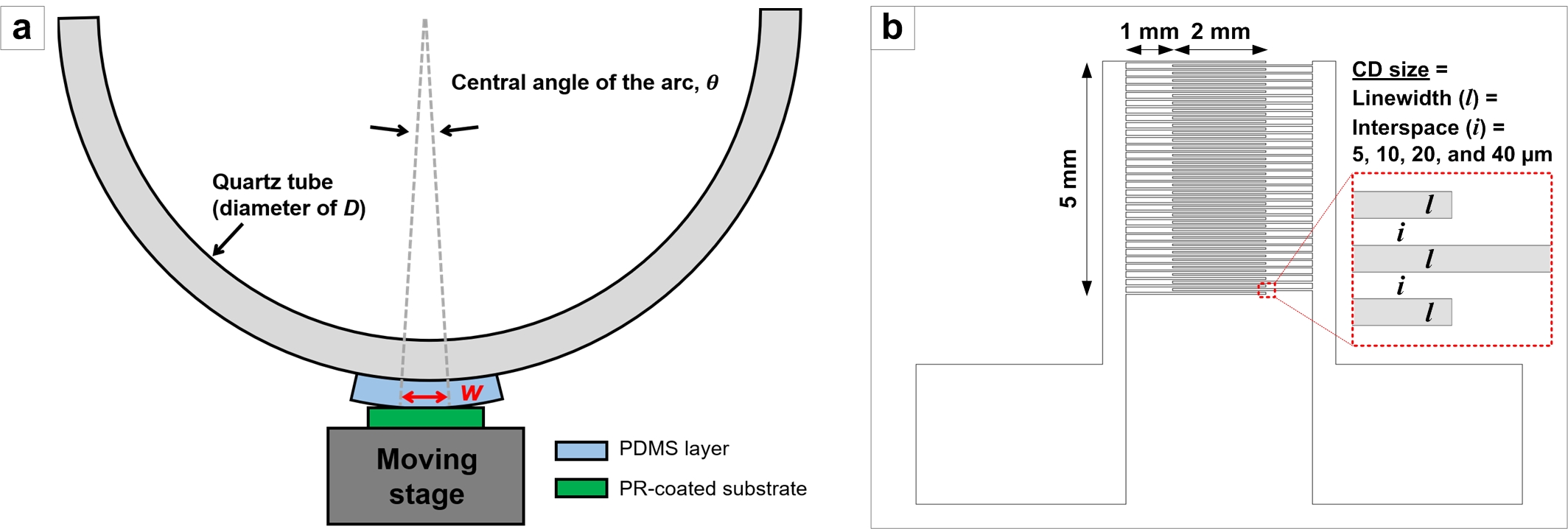


**Figure S2**. (a) Diagram of the UV exposure slit width (*w*) in the PRL system, given by *θD*/2, where *θ* and *D* are the central angle of the arc and the quartz tube diameter, respectively. When the *w*=1 mm slit is used in the *D*=150 mm tube, *θ* is determined to be 0.0133 rad, which has a negligible effect on light divergence across the slit width. (b) CAD drawing of the interdigitated (IDT) microelectrode design. The linewidth and interspaces (referred to as critical dimension (CD) sizes) are varied at 5, 10, 20, and 40 µm for a parametric investigation, as discussed in the main text.


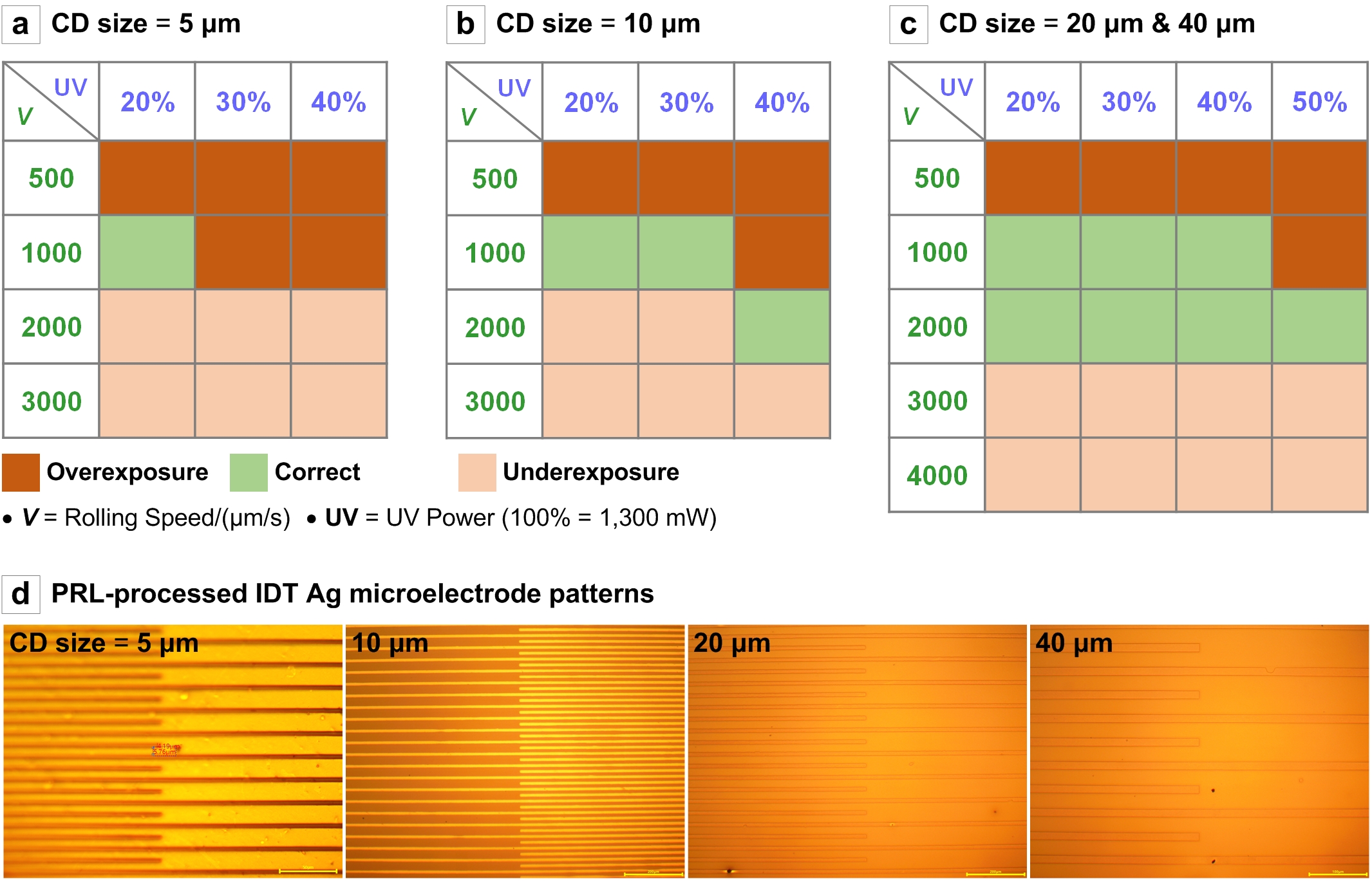


**Figure S3**. (a-c) Tabulated PRL parameters (rolling speed and UV power) leading to UV overexposure, correct UV exposure, and UV underexposure, for (a) 5 µm, (b) 10 µm, and (c) 20 µm IDT Ag microelectrode patterning. (d) Representative OM images of correctly fabricated IDT Ag microelectrode structures with various CD sizes.


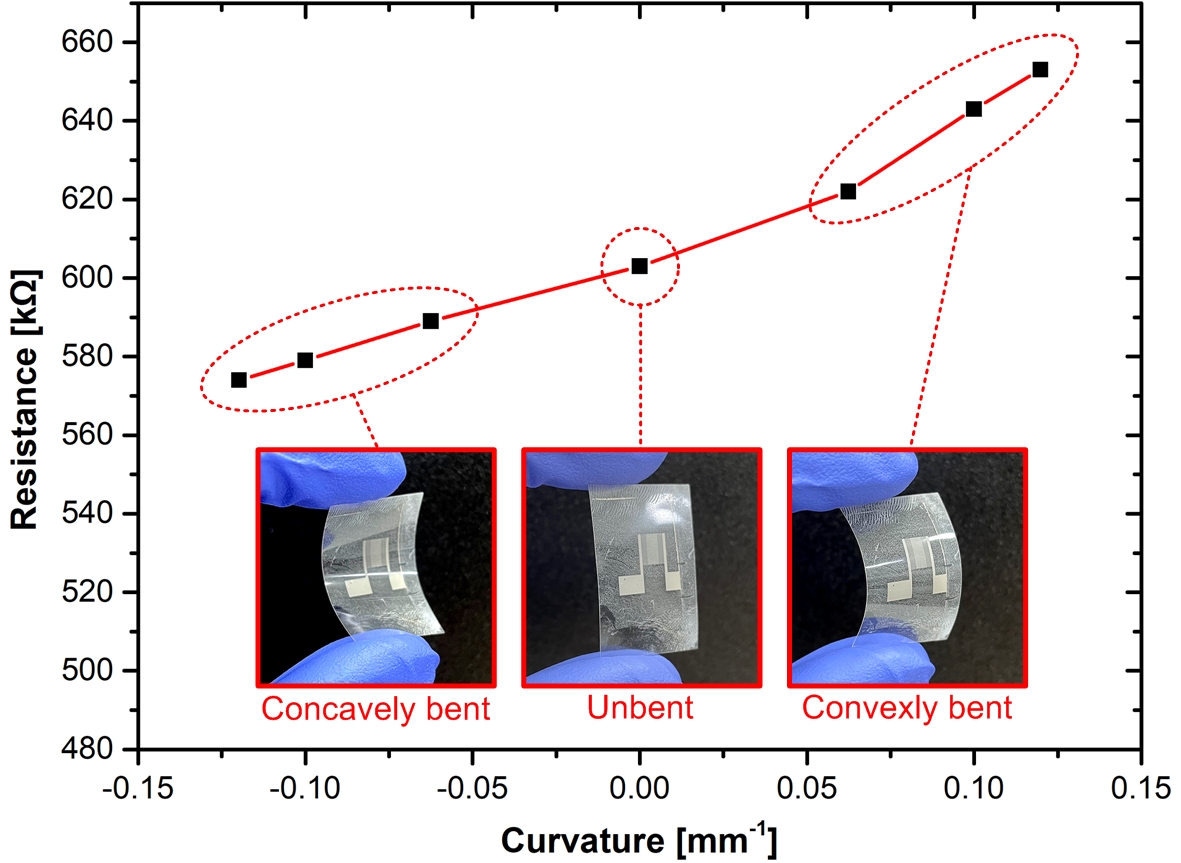


**Figure S4**. Resistance of the flexible ZNW/IDT device measured at various bending curvatures. The negative and positive curvature values denote the concave and convex bending statuses, respectively; the insets show optical photographs of the ZNW/IDT device under various bending statuses.


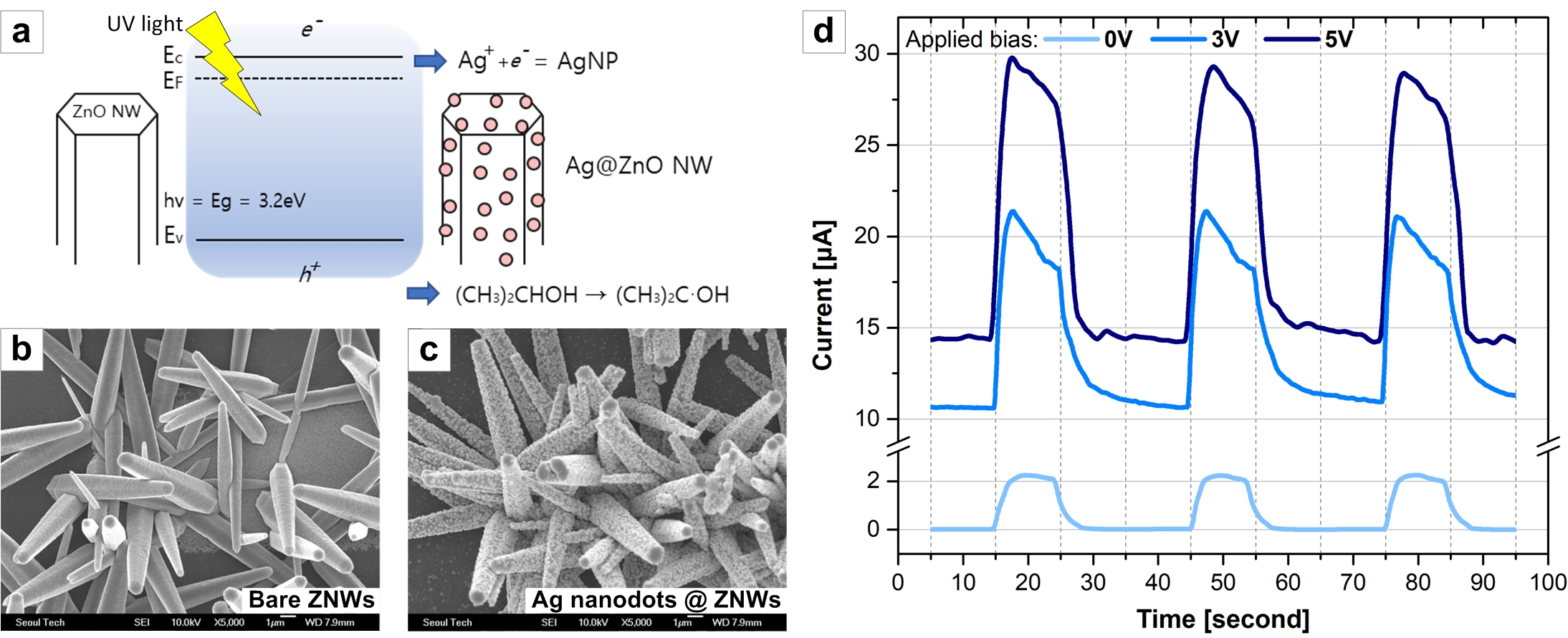


**Figure S5**. (a) The mechanism of the room-temperature photoreduction of Ag nanodots on the ZnO surface. The photogenerated free electrons can chemically reduce the Ag ions into the Ag nanodots at room temperature and high speed. Representative SEM images of (b) bare ZNWs, (c) ZNWs coated with Ag nanodots. (d) Photocurrents (*versus* time) of the Ag nanodot-coated ZNW/IDT structure, measured at various applied biases of 0 V, 3 V, and 5 V under pulsed UV illumination at a fixed intensity of 500 mW/cm^2^.
